# Association of Body Mass Index with Cause Specific Deaths in Chinese Elderly Hypertensive Patients: Minhang Community Study

**DOI:** 10.1371/journal.pone.0071223

**Published:** 2013-08-13

**Authors:** Yan Wang, Yajuan Wang, Yuesheng Qain, Jin Zhang, Xiaofeng Tang, Junlei Sun, Dingliang Zhu

**Affiliations:** 1 State Key Laboratory of Medical Genomics, Ruijin Hospital, Shanghai Jiaotong University School of Medicine, Shanghai, China; 2 Research Center for Hypertension Management and Prevention in Community, Shanghai Institute of Hypertension, Shanghai Jiaotong University School of Medicine, Shanghai, China; 3 Xinzhuang Community Health Service Center, Shanghai, China; Pennington Biomedical Research Center, United States of America

## Abstract

**Background:**

Most studies have suggested that elevated body mass index (BMI) was associated with the risk of death from all cause and from specific causes. However, there was little evidence illustrating the effect of BMI on the mortality in elderly hypertensive patients in Chinese population.

**Methods:**

The information of 10,957 hypertensive patients at baseline not less than 60 years were from Xinzhuang, a town in Minhang district of Shanghai, was extracted from the Electronic Health Record (EHR) system. All study participants were divided into eight categories of baseline BMI (with cut-points at 18, 20, 22, 24, 26, 28 and 30 kg/m^2^). Relative hazard ratio of death from all cause, cardiovascular and non-cardiovascular cause by baseline BMI groups were calculated, standardized for sex, age, smoking, drinking, physical activity, systolic blood pressure, history of cardiovascular disorders, serum lipid disturbance, diabetes mellitus and antihypertensive drug treatment.

**Results:**

During follow up (median: 3.7 years), 561 deaths occurred. Underweight (BMI<18 kg/m^2^) was associated with significantly increased mortality from all cause mortality (OR: 2.00; 95% CI: 1.43–2.79) and non cardiovascular mortality (OR: 2.76; 95% CI: 1.87–4.07), but not with cardiovascular mortality. For the cause specific analysis, the underweight was associated significantly with neoplasms (OR: 2.15; 95% CI: 1.16–4.00) and respiratory disorders (OR: 3.41; 95% CI: 1.64–7.06). The results for total mortality and specific cause mortality were not influenced by sex, age and smoking status.

**Conclusion:**

Our study revealed an association between underweight and increased mortality from non-cardiovascular disorders in elderly hypertensive patients in Chinese community. Overweight and obesity were not associated with all cause or cause specific death.

## Introduction

The prevalence of hypertension in China keeps increasing, until 2010, more than 200 million people suffer high blood pressure and it has become the leading preventable cause of death among adults 40 years of age and older [1]. At the same time, obesity and overweight have become a serious public health problem accompanying with the changes in dietary habits and physical activity[2]–[4]. Body-mass index (BMI) is a reasonably good measure of general adiposity [5], and raised BMI is an established risk factor for several disorders, including hypertension [6], diabetes [7], and so on. Although obesity is clearly a risk factor for developing hypertension, once hypertension has been established, the correlation of obesity with total mortality and cardiovascular mortality is still unclear. The optimal BMI and the effects of being either underweight or overweight on the risk of death are controversial. At the same time, there is long-standing disagreement regarding the effect of age on the association between underweight and all-cause mortality[8]–[15]. This inconsistency may be partly due to inadequate adjustment for several confounders such as cigarette smoking, alcohol consumption, physical activity, socioeconomic status and underlying diseases.

As for most studies of the association between BMI and death in hypertension patients have been conducted in populations of European or American origin [16], [17], it is uncertain whether the findings of these studies can be applied to other groups. Chinese populations have a relatively higher percentage of body fat for a given BMI than do whites [18]. The dose–response relationship between BMI and the overall risk of death among hypertensive patients in China remains unclear. Therefore, to investigate the overall and specific risk of death associated with body weight in elderly hypertensive patients, we examined the association between BMI and cause specific death rates in a hypertension management database derived from electronic health records in Chinese community hospitals.

## Methods

### Minhang EHR System

This research was designed as a retrospective study of Xinzhuang community population in Shanghai, China. Xinzhuang was one of 13 towns in Minhang District, covering an area of 19.53 km^2^ and with a population of about 278,000, which was a recent urbanized area.

There were 13 town- and 5 district- hospitals in Minhang district. The primary care of hypertension was managed locally in each town hospital, and all these hospitals used the same electronic health records (EHR) of the Mihang electronic primary care system implemented in 2007. The electronic system improved the primary healthcare by integrating the outpatient clinic activity and periodic follow-up, connecting all primary care centers to town- and district- hospitals within Minhang. In the system, each patient owned a health card with unique personal identification number, which allowed him/her to access their own EHR that containing all clinical and laboratory data, as well as tracking electrocardiography and cardiac and vascular ultrasonographic images. It was composed of special modules for chronic diseases management, including hypertension, diabetes mellitus and malignant tumors. At the first visit, all the hypertensive patients were classified automatically as low, moderate, high and very high risk of cardiovascular morbidity and mortality in the coming 10 years, according to the blood pressure (BP) level, risk factors and established cardiovascular or renal disease. Then, the periodic follow-up was performed according the risk stratification groups that the patients in high and very high risk group should be followed-up every month, moderate risk group every two months, and low risk group every three months. Meanwhile, the cardiovascular event and death were recorded and confirmed by the physicians.

### Study Population

All data used for this study were extracted from the EHR from Jan. 1, 2007, to Sep 27, 2011 in Xinzhuang town hospital. A total of 10,957 hypertensive patients no less than 60 years old were included in the study (5,215 men and 5,742 women).

Hypertensive status was based on systolic blood pressure (SBP) more than 140 mmHg or diastolic blood pressure (DBP) more than 90 mmHg, or taking antihypertensive medication. Three BP readings of different days, measured with the use of a mercury sphygmomanometer according to a standard protocol, were obtained after the participant had been sitting quietly for five minutes. BMI was calculated as self-reported weight in kilograms divided by the square of height in meters. All study participants were divided into eight categories of baseline BMI (with cut-points at 18, 20, 22, 24, 26, 28 and 30 kg/m^2^). We used the term underweight for BMI less than 18.0 kg/m^2^, overweight for BMI over 24 kg/m^2^ and obesity for BMI over 28 kg/m^2^. Diabetes was diagnosed according to American Diabetes Association criteria. Hyperlipidemia status was based on total cholesterol ≥5.7 mmol/L or low density lipid cholesterol >3.3 mmol/L, or high density lipid cholesterol <1.0 mmol/L. Cigarette smoking was defined as smoking at least one cigarette per day for one or more years. Alcohol drinking was defined as those drunk two or more days of every week during the past 6 months. The cardiovascular history was obtained through questionnaire.

### Ethics Statement

This study was approved by the Ethics Committee of Ruijin Hospital. Considering it was about the past information stored in the hospital database, written consent was specifically waived by the approving Ethics Committee.

### Ascertainment of Death

We ascertained vital status until December 16, 2011, via EHR. Furthermore, all the information of death was confirmed by the death documents provided by Minhang Centers for Disease Control and Prevention. End points considered in the present analysis were death from all causes, cardiovascular (International Classification of Diseases, 10th Revision [ICD-10] codes “I”) and non-cardiovascular mortality, mortality from cerebral hemorrhage (ICD-10 code I60–62), cerebral infarction (ICD-10 code I63),cardiac disorders (ICD-10 codes I05, I11, I20–I25, I34, I35, I38, I46–I50, I71, I74, I77, and I99), neoplasms (ICD-10 codes C00–D48), diseases of the respiratory system (ICD-10 codes “J”), diabetes related (ICD-10 codes “E10–E14”), digestive (ICD-10 codes “K”), and accident (ICD-10 codes “X”). Thus, cardiovascular mortality includes mortality from all stroke and cardiac events.

### Statistics

For database management and statistical analysis, we used SPSS software (version 13.0; SPSS Inc., Chicago, Illinois, USA). Descriptive statistics for patients among groups by BMI category were compared using a Pearson Chi-square test of significance. Survival curves by Kaplan-Meier survival function was used to estimate for mortality across different BMI groups. Cox proportional hazards regression analysis was used to calculate the hazard ratios (HRs) and 95% confidence intervals (CIs) for all-cause and specific-cause mortality according to BMI category. Stratified analyses were conducted according gender, age and smoking status. Because the relation between mortality and BMI was not linear, we used the deviation from the population mean coding to estimate the risk in BMI categories. This approach expressed the risk in each group relative to the overall risk in the whole study population and allows computation of CIs for the hazard ratio in each group without definition of an arbitrary reference group. Initial regression models included adjustment for sex and age. The fully adjusted model included smoking, drinking, physical activity, SBP, history of cardiovascular disorders, serum lipid disturbance, diabetes mellitus and antihypertensive drug treatment. All P values were 2-tailed, and a P value of <0.05 was considered statistically significant.

## Results

Until Dec 16, 2011, 1,871 patients dropped off, and the remaining 9,186 hypertensive patients were included in the final analysis, including 4,361 men and 4,825 women. Comparing with the subject included, participants who failed to complete the follow-up were older (72.0±7.8), with lower antihypertensive treatment rate (88.9%) and physical activity (25.1%). The baseline characteristics of the study population according to BMI categories were presented in Table 1. Overall, the mean age was 70.7 years, with a range from 60 to 97 years. 47.5% of the hypertensive patients enrolled was male. Highest death rate was observed in those with lowest BMI (20.1%). The death rate in the group with BMI over 22 kg/m^2^ was only 5%, which was approximately one half that under 22 kg/m^2^. The patients with lower BMI were older, and showed lower BP and physical activity. There was no significant difference found among groups in current smoking, drinking, diabetes and antihypertensive treatment.

**Table 1:** Baseline characteristics of the community hypertensive patients by category of body-mass index.

Characteristics	Body mass index (kg/m^2^)								*P*
	<18	18–20	20–22	22–24	24–26	26–28	28–30	≥30	
N	194	672	1560	2442	2103	1313	570	332	
Death (n, %)	39 (20.1)	63 (9.4)	119 (7.6)	143 (5.9)	98 (4.7)	56 (4.3)	30 (5.3)	13 (3.9)	***<0.001***
SBP (mmHg)	133.2±7.7	133.3±8.8	133.3±9.7	133.5±8.2	133.9±8.7	134.0±9.4	134.8±8.2	134.7±11.3	***0.002***
DBP (mmHg)	80.5±5.5	81±4.8	80.8±5.5	81.3±4.7	81.6±4.7	82.0±5.3	82.2±4.8	81.9±6.7	***<0.001***
Age (y)	75.6±8.4	73.4±8.3	71.9±7.7	70.6±7.2	70.0±7	69.5±7	69.7±7.1	69.4±6.4	***<0.001***
Male (n,%)	91(46.9)	287(42.7)	686(44)	1227(50.2)	1055(50.2)	655(49.9)	240(42.1)	120(36.1)	***<0.001***
Smoking (n,%)	18(9.3)	55(8.2)	170(10.9)	261(10.7)	240(11.4)	143(10.9)	55(9.6)	24(7.2)	0.149
Drinking (n,%)	6(3.1)	22(3.3)	54(3.5)	90(3.7)	107(5.1)	51(3.9)	24(4.2)	12(3.6)	0.194
Lack of physical activity (n,%)	151(77.8)	524(78)	1128(72.3)	1650(67.6)	1418(67.4)	904(68.8)	392(68.8)	239(72)	***<0.001***
Diabetes mellitus (n,%)	15(7.7)	63(9.4)	170(10.9)	291(11.9)	251(11.9)	155(11.8)	79(13.9)	45(13.6)	0.123
Serum lipid disorder (n,%)	6(3.1)	15(2.2)	33(2.1)	65(2.7)	72(3.4)	42(3.2)	22(3.9)	24(7.2)	***<0.001***
Cardiovascular disorders (n,%)	38(19.6)	88(13.1)	198(12.7)	267(10.9)	240(11.4)	151(11.5)	79(13.9)	37(11.1)	***0.013***
Antihypertensive treatment (n,%)	185(95.4)	647(96.3)	1508(96.7)	2357(96.5)	2045(97.2)	1277(97.3)	555(97.4)	327(98.5)	0.295
Antihypertensive number (n)	1.05±0.31	1.07±0.29	1.08±0.31	1.09±0.34	1.09±0.35	1.11±0.37	1.10±0.36	1.14±0.42	***0.004***

Data are No. (%) or mean±SD. SBP and DBP indicate systolic and diastolic blood pressures. P values are for the difference among the eight groups. P values less than 0.05 are shown in bold and italics.

During follow up (median: 3.7 years), 561 deaths occurred. Mortality included 242 cardiovascular deaths, with 142 strokes and 100 cardiac deaths. Stroke deaths were because of cerebral infarction in 91 subjects, cerebral hemorrhage in 40 subjects. Cardiac mortality included chronic coronary heart disease (n = 61), myocardial infarction (n = 18), heart failure (n = 3), and various other cardiac disorders (n = 18). Non-cardiovascular deaths (n = 319) resulted from neoplasms (n = 178), diseases of the respiratory system (n = 61), diabetes related (n = 32), diseases of the digestive system (n = 8), accident (n = 14) and various other diseases (n = 26).

**Figure 1:**
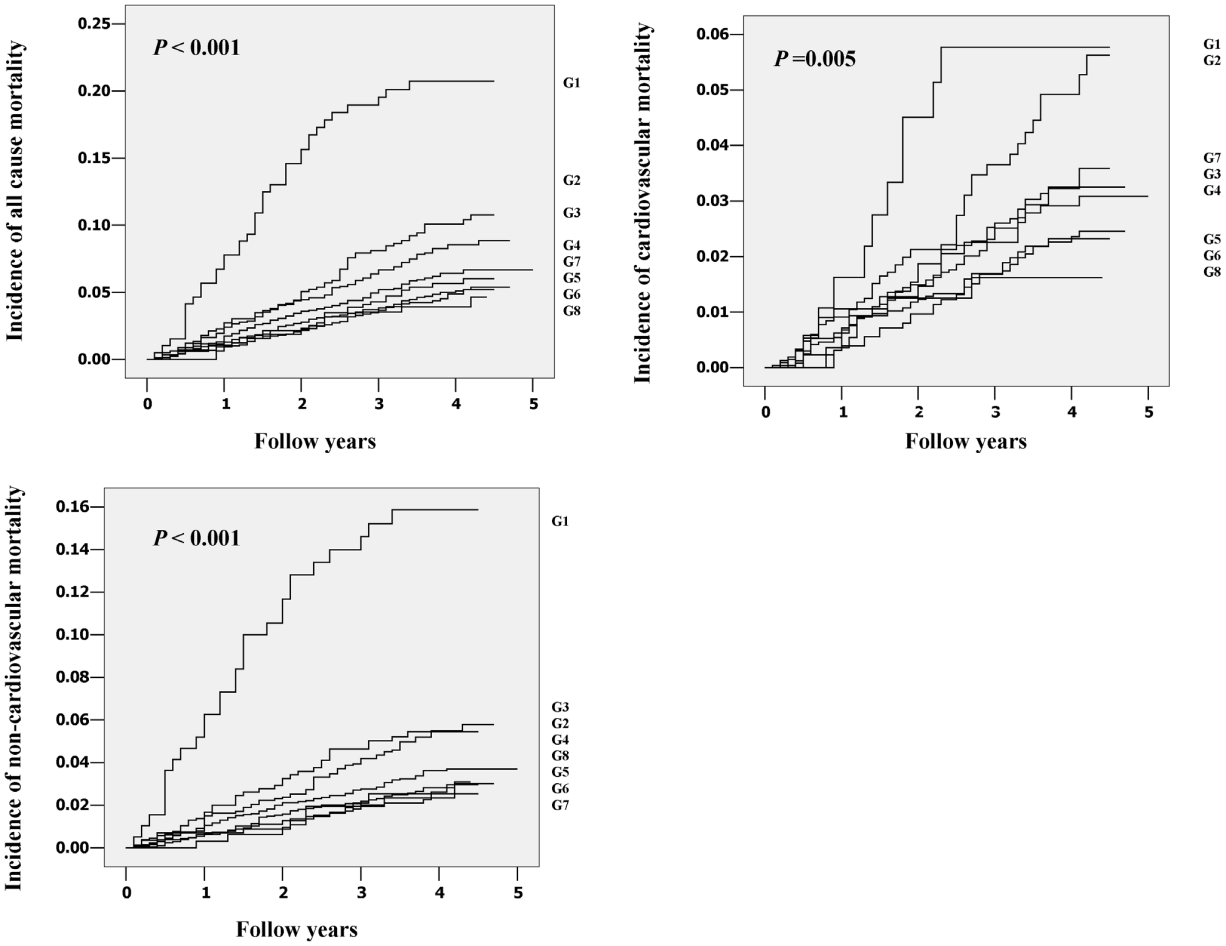
Cumulative incidence of all causes mortality, cardiovascular mortality and non-cardiovascular mortality across eight groups of body mass index (BMI). G1 to G8 indicate ascending BMI groups. Cutoff points were 18, 20, 22, 24, 26, 28,30 kg/m^2^.

**Figure 2:**
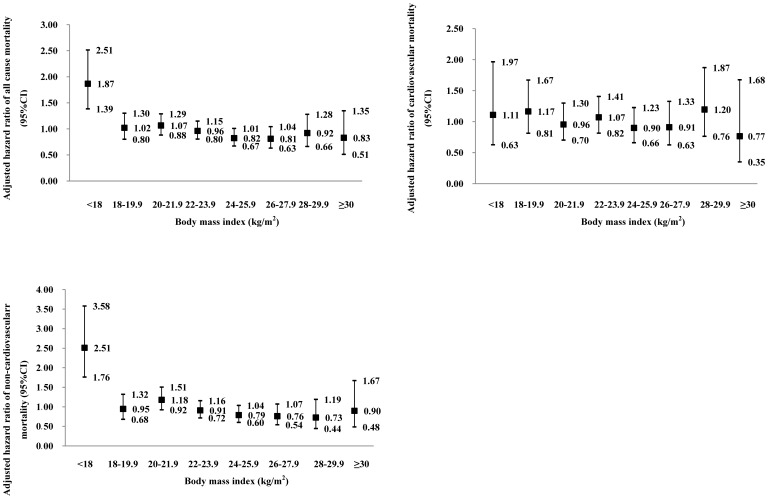
Multivariable-adjusted hazard ratios (95% CI) for all causes mortality, cardiovascular mortality and non-cardiovascular mortality across eight groups of body mass index (BMI). The hazard ratio express the risk in the BMI group compared with the average risk in the whole study population, which were adjusted for sex, age, smoking, drinking, physical activity, systolic blood pressure, history of cardiovascular disorders, serum lipid disturbance, diabetes mellitus and antihypertensive drug treatment.

Cumulative incidence of all causes mortality, cardiovascular mortality and non-cardiovascular mortality across eight groups of BMI was shown in Figure 1. In Cox regression, the prognostic significance of the lowest BMI group was analyzed with adjustments applied for sex and age in basic model and for smoking, drinking, physical activity, SBP, history of cardiovascular disorders, serum lipid disturbance, diabetes mellitus and antihypertensive drug treatment in the fully adjusted model. The adjusted HRs for underweight (BMI<18 kg/m^2^) on death from cause-specific mortality were shown in Table 2. The all cause and non-cardiovascular mortality were elevated in the lowest BMI group, with a HR of 2.00 (95% CI: 1.43–2.79) for total death and of 2.76 (95% CI: 1.87–4.07) for non-cardiovascular death. To investigate further, cardiovascular deaths were subdivided into cardiac and cerebrovascular death, and non-cardiovascular were subdivided into deaths from neoplasm, respiratory disease, diabetes related disease, digestive disease and accident. The results indicated that underweight was associated with excess death from neoplasms (HR: 2.15; 95% CI: 1.16–4.00) and respiratory disorders (HR: 3.41; 95% CI: 1.64–7.06). However, for cardiovascular diseases, underweight was not associated with excess mortality, even if stroke and heart disease related death were analyzed respectively. Figure 2 shows the fully adjusted hazard ratios for all-cause, cardiovascular and non-cardiovascular mortality by eight groups of BMI. Only in the lowest BMI group was the risk significantly higher than the average risk in the whole population, whereas in the 22–23.9 kg/m^2^ group, the risk was approximately half and one third for all cause and non-cardiovascular mortality (Figure 2). We also checked the consistency of our results for total mortality and all cardiovascular events according to various baseline characteristics (Table 3). After the subjects were subdivided according to quintiles of age, the magnitude of the association was similar among groups, except for the youngest group which included too few underweight patients to calculate the hazard ratio. The results for men and women were similar to the results of combined analyses of data from men and women. However, in the current smokers group, the association of underweight with excess death from total cause and non-cardiovascular death was not significant, whereas the predictive effect of underweight for death in current non-smokers was similar to the total population.

**Table 2:** Hazard ratios for cause-specific mortality according joint categories of body mass index.

	Body mass index<18 kg/m^2^	
Mortality	Adjusted	Fully adjusted
All causes (561)	2.04 (1.47–2.84)¶	2.00 (1.43–2.79)¶
Cardiovascular (242)	1.16 (0.62–2.20)	1.12 (0.59–2.12)
Cardiac (100)	1.76 (0.76–4.05)	1.81 (0.78–4.19)
Cerebrovascular (142)	0.77 (0.28–2.09)	0.70 (0.26–1.90)
Infarction (91)	0.56 (0.14–2.32)	0.51 (0.12–2.08)
Hemorrhage (40)	0.73 (0.10–5.33)	0.70 (0.11–1.84)
Non-cardiovascular (319)	2.76 (1.88–4.07)¶	2.76 (1.87–4.07)¶
Neoplasm (178)	2.18 (1.17–4.04)*	2.15 (1.16–4.00)*
Respiratory (61)	3.61 (1.75–7.43)§	3.41 (1.64–7.06)§
Diabetes related (32)	1.97 (0.46–8.36)	2.73 (0.64–11.71)
Digestive (8)	3.14 (0.37–26.28)	3.60 (0.43–30.49)
Accident (14)	3.37 (0.73–15.48)	3.50 (0.77–15.93)

Hazard ratios (95% CI) indicates the risk of body mass index (BMI) <18 kg/m^2^ compared with the risk in all the other BMI groups. The adjusted model includes sex and age. The fully adjusted model includes smoking, drinking, physical activity, systolic blood pressure, history of cardiovascular disorders, serum lipid disturbance, diabetes mellitus and antihypertensive drug treatment additionally.

* p<0.05,

§ p<0.01,

¶ p<0.001.

**Table 3:** Multivariable adjusted hazard ratios for all causes mortality, cardiovascular mortality and non-cardiovascular mortality of body mass index (BMI) <18 kg/m^2^ in subgroup analysis.

		All causes mortality		Cardiovascular mortality		Non-cardiovascular mortality	
Mortality	At risk, n	Deaths, n	BMI <18 kg/m^2^	Deaths, n	BMI <18 kg/m^2^	Deaths, n	BMI <18 kg/m^2^
All participants	9186	561	2.00 (1.43–2.79)¶	242	1.12 (0.59–2.12)	319	2.76 (1.87–4.07)¶
Women	4825	256	1.86 (1.16–3.09)*	120	1.42 (0.62–3.25)	136	2.28 (1.19–4.36)*
Men	4361	305	2.15 (1.39–3.34)§	122	0.87 (0.32–2.39)	183	3.10 (1.89–5.08)¶
60–63.4 years	1835	26	–	12	–	14	–
63.4–67.6 years	1842	30	5.78 (1.33–25.2)*	9	7.14(0.84–60.7)	21	4.77(0.61–37.08)
67.6–72.4 years	1834	72	3.41 (1.05–11.1)*	28	–	44	5.77 (1.73–19.20)§
72.4–77.2 years	1840	127	2.94 (1.42–6.10)§	59	–	68	7.31 (3.40–15.73)¶
≥77.2 years	1835	306	1.82 (1.21–2.74)§	134	1.37(0.69–2.73)	172	2.17 (1.31–3.62)§
Non-smokers	8220	497	2.06 (1.45–2.93)¶	214	1.06 (0.52–2.15)	283	2.92 (1.94–4.39)¶
Smokers	966	64	1.89 (0.68–5.26)	28	2.90 (0.61–13.9)	36	1.39 (0.36–5.40)

The Cox regression model were adjusted for sex, age, smoking, drinking, physical activity, systolic blood pressure, history of cardiovascular disorders, serum lipid disturbance, diabetes mellitus and antihypertensive drug treatment additionally. Subgroups of age were determined according to quintiles.

* p<0.05,

§ p<0.01,

¶ p<0.001.

## Discussion

In this study, we covered a large part of the hypertension patients no less than 60 years resided in Xinzhuang community. According to the data of national census in 2010, people aged 60 and older accounted for 12.29 percent of the 278,000 residents in Xinzhuang. In addition, the prevalence of hypertension among elderly people was about 49.73% [19]. Therefore, over 64% of 16,990 elderly hypertensive patients lived in this area were included in this study.

Our results showed that increased total and non-cardiovascular mortality associated with underweight (<18 kg/m^2^) in elderly hypertensive patients. The lowest all-cause mortality was found among participants with a BMI over 24 kg/m^2^ in both sexes. Underweight was associated with about 100% higher all-cause mortality (176% for non-cardiovascular and 115% for neoplasm), and no specific cause of cardiovascular death was associated with underweight or overweight. In the specific cause analysis, we found that excess death from neoplasms and respiratory disorders was owing to underweight. Such result was in accordance with the findings from the previous studies. In 2009, Whitlock et al analyzed the baseline BMI versus mortality in 57 prospective studies with 894,576 participants, mostly in Western Europe and North America. They found that overweight was associated overall mortality, and death caused by vascular, diabetic, renal, and hepatic, neoplastic and respiratory disease, and underweight was associated inversely with overall mortality, especially associated with death related to respiratory disease and lung neoplasms [20].

A large amount of previous studies conducted in Western populations have reported a U-shape or J-shape association between BMI and all-cause mortality [10], [20]. On the contrary, some researchers emphasized the overwhelming effect of obesity [11] or leanness [16], [21]on the excess of death. What the age structure of the population was in these studies and whether or not subgroup analysis performed according to ages might be one of the major reasons for the controversial results. Flegal and colleagues have analyzed the data from National Health and Nutrition Examination Survey (NHANES) I-III with underlying cause of death information for 2.3 million adults 25 years and older in US [12], [22]. They reported that only underweight increased the risk of mortality in the group over 70 years old. In addition, the Ohsaki Cohort Study, which included 43,972 Japanese participants aged 40 to 79 years, showed a significantly increased risk of mortality in underweight elderly men and women [23]. Our study focusing on the elderly population also found that underweight but not overweight or obesity would increase risk of death.

The accompanying disorders might also undermine the replication of association of body weight with mortality. The study by Adams et al examined data from National Institutes of Health–AARP cohort with 527,265 US men and women who were 50 to 71 years old at enrollment. Initial analyses showed an increased risk of death for both the highest and lowest categories of BMI, regardless of sex or age. When the analysis was restricted to healthy people who had never smoked, the risk of death was associated with only overweight [11]. It indicated that the underlying disorders and accompanying unhealthy lifestyle might influence the relationship between underweight and death. Our results showed that underweight could increase the risk of all-cause death in hypertension patients. Studies that assessed the relation between BMI and mortality in patients with coronary disease and heart failure have shown a similar pattern [24]. Romero-Corral and colleagues performed a meta-analysis of all available cohort studies examining the association between body weight and total mortality, cardiovascular mortality, and other cardiovascular events, including over 250 000 patients with established coronary-artery disease [21]. For patients with established coronary artery disease, low BMI was strongly associated with increased long-term risk of death and cardiovascular events relative to normal weight. Overweight patients were consistently associated with a better survival and fewer cardiovascular events than normal BMI patients. In addition, systematic reviews of patients in intensive care have also shown that low-normal BMI values are associated with higher in-hospital mortality [25]. It is undoubted that obesity is an independent risk factor for cardiovascular disorders, including heart failure, coronary artery disease and hypertension. However, the prognostic significance of obesity in the setting of established cardiovascular disorders is not clear. Observational data suggested a protective effect of obesity, which has been termed the obesity paradox or reverse epidemiology [26]. Several potential explanations for the obesity paradox existed. First, fat storage might be protective in some individuals exposed to acute insults or to chronic wasting, resources that people with low-normal BMI did not have [27]. Second, BMI might not adequately reflect adiposity. BMI was not able to discriminate between an excess in body fat and increments in lean mass. In addition, studies had shown that for a given BMI, Asians generally had a higher percentage of body fat than do Europeans [28]. Third, selection bias might confuse the results. Most patients with obesity might be presenting earlier and acquired more attention and treatment from the doctors.

In addition, the population of our study was derived from the community, therefore the BP was relatively low and the level of each group was nearly identical. This partly explained why there was little difference in cardiovascular mortality (albeit a trend to increased mortality in the low BMI group) yet increases in non-cardiovascular related mortality.

Several limitations of our study should be considered. First, we used self-reported BMI at baseline instead of measurement. But, it was reported that the correlation between BMI based on self-reported and that based on measured height and weight was typically greater than 0.9 [29]. Second, we did not exclude the subjects with a baseline diagnosis of coronary heart disease, stroke, or neoplasm, which might possibly induce reverse causation. But we had excluded the smokers, which had been indicated as the one strong reason for reverse causation, and got the similar result from whole population. Third, obesity was only estimated by BMI. It was suggested that both general adiposity and abdominal adiposity were associated with the risk of death, which supported the use of waist circumference or waist-to-hip ratio in addition to BMI in assessing the risk of death [30].

In conclusion, this study revealed an association between underweight and the risk of death in elderly hypertension patients in Chinese community. Overweight and obesity were not associated with all cause or cause specific death. Studies based on abdominal obesity and other exact measurements of body fat were suggested verifying the relationship between underweight and excess death. In addition, the further long-term cohort study was warranted to elucidate the concrete effect of baseline BMI influenced the cause specific mortality.
